# How useful is the Making Every Contact Count Healthy Conversation Skills approach for supporting people with musculoskeletal conditions?

**DOI:** 10.1007/s10389-022-01718-y

**Published:** 2022-05-04

**Authors:** Amelia Parchment, Wendy Lawrence, Em Rahman, Nick Townsend, Elaine Wainwright, David Wainwright

**Affiliations:** 1 Department for Health, University of Bath, Bath, England BA2 7AY UK; 2 MRC Lifecourse Epidemiology Unit, University of Southampton, Southampton, England SO16 6YD UK; 3 NIHR Southampton Biomedical Research Centre, University Hospital Southampton NHS Foundation Trust, Southampton, England SO16 6YD UK; 4 Public Health Workforce Development, Southern House, Health Education England, Winchester, England SO21 2RU UK; 5 Aberdeen Centre for Arthritis and Musculoskeletal Health (Epidemiology Group), School of Medicine, Medical Sciences and Nutrition, University of Aberdeen, Aberdeen, UK

**Keywords:** Making every contact count, Healthy conversation skills, Very brief intervention, Brief intervention, Physiotherapist, Musculoskeletal health, Theoretical domains framework

## Abstract

**Aim:**

To explore the current use and perceptions of the Wessex model of Making Every Contact Count (MECC), incorporating Healthy Conversation Skills (HCS), focussing specifically on physiotherapists supporting people living with musculoskeletal conditions.

**Methods:**

A mixed method, sequential explanatory design was employed. This article reports the first phase of the study, in which an online questionnaire was administered, consisting of items relating to perceived acceptability, appropriateness, feasibility, sustainability, and uptake of MECC HCS. Barriers and facilitators to MECC HCS delivery were additionally explored and mapped to the Theoretical Domains Framework.

**Results:**

Seventy-one professionals responded, including 15 physiotherapists supporting people with MSK conditions. Across professional groups, MECC HCS was found to be highly acceptable, appropriate, and feasible. A significant interaction between perceived sustainability of MECC HCS and the location in which professionals worked was observed. Physiotherapists reported using their MECC HCS at least daily; however, there were discrepancies between the number of their patients they believed could benefit from behaviour change intervention, and the number to whom they reported actually delivering MECC HCS. Perceived barriers and facilitators to MECC HCS implementation mapped mostly to ‘Environmental Context and Resources’ on the Theoretical Domains Framework.

**Conclusions:**

The Wessex model of MECC is a promising brief or very brief intervention for physiotherapists supporting individuals with musculoskeletal conditions. Barriers associated with the sustainability of the intervention within organisations must be addressed in order to enhance future implementation. Further rollout of this intervention may be beneficial for meeting the goals of the NHS and Public Health England in prevention of chronic MSK conditions and promotion of musculoskeletal health.

## Introduction

### Making Every Contact Count

The health risk factors related to chronic and non-communicable diseases (NCDs) are well documented (Budreviciute et al. 2020; Nyberg et al. 2018; Forouzanfar et al. 2016). Lifestyle factors, such as alcohol consumption, stress, diet, smoking, and physical activity, combined with wider, socio-economic determinants of health (eg., education, employment, and income) are strongly associated with the development of cardiovascular and respiratory diseases, cancers, diabetes, musculoskeletal disorders, and other NCDs. Many cases are preventable and some risk factors amenable to change (Mikkelsen et al. 2019).

Making Every Contact Count (MECC) is a behaviour change initiative that has formed part of the UK’s National Health Service’s (NHS) plan to embed NCD prevention and health promotion into the everyday practice of all staff, including those who are non-specialist and have little to no experience in public health [Public Health England (PHE), NHS England and Health Education England (HEE) 2016]. Drawing upon established behavioural science that recognises the range of individual, social, cultural, and wider socio-economic factors that can influence behaviour (Michie et al. 2011), MECC aims to utilise the multiple interactions (and thus, health promotion opportunities) health and social care practitioners have with the public. Staff are trained to deliver very brief or brief interventions (V/BI), targeting lifestyle behaviours, ranging from simply raising awareness of health risk factors and signposting to relevant services, to supporting behaviour change through discussion and encouragement. A MECC very brief intervention can be delivered in as little as 30 seconds, and a brief intervention in 5 to 15 minutes. They aim to ‘enable a positive change in an individual by increasing their psychological capability to undertake a behaviour change’ (PHE, NHS England, and HEE 2016). Since MECC’s introduction within the NHS, the initiative has extended to local authorities, private, and third sectors.

Despite its vast rollout, there is no universal method of training for MECC nor a consistent approach to its delivery. Rather, different regions and organisations have adopted their own ways of adhering to policies relating to public health, prevention, and MECC as an evidence-based intervention (Haighton et al. 2021; Parchment et al. 2021). Research suggests that MECC training increases the confidence and competence of trainees in delivering the V/BI, and is considered mostly acceptable (Webster 2018; Dewhirst and Speller 2015; Chisholm et al. 2019). Other studies have highlighted that MECC is not delivered routinely, even when it is believed that patients could benefit (Keyworth et al. 2018a, b), and staff perceive there to be barriers to successful implementation (Chisholm et al. 2019; Elwell et al. 2014; Nelson et al. 2013; Keyworth et al. 2019; Mulroe et al. 2017). The limited evidence base highlights that MECC research is still in its infancy, and more evaluation for staff and service users across regions is warranted to assess the impact of its implementation on a wider scale.

### Healthy Conversation Skills

Healthy Conversation Skills (HCS) is the main training component of Health Education England Wessex’s approach to MECC (see Appendix 1 for training skills) and, as a standalone intervention, has demonstrated effectiveness and acceptability for both staff delivering and service users receiving it (Dewhirst and Speller 2015; Lawrence et al. 2016; Lawrence et al. 2020; Black et al. 2014; Jarman et al. 2019; Adam et al. 2020; Baird et al. 2014). Competence and confidence in using learned skills to support behaviour change have been demonstrated 1 year post-training, suggesting long-term positive impacts of implementation (Baird et al. 2014; Lawrence et al. 2016). As a person-centred approach, HCS aims to empower individuals to change their behaviours by increasing their self-efficacy; a construct central to Social Cognitive Theory (Bandura 1986, 1997). Self-efficacy reflects an individual’s belief in their capability to perform specific behaviours necessary to achieve their goals. Those delivering the intervention employ behaviour-change techniques that are known to encourage self-efficacy, such as problem solving and goal setting (Michie et al. 2011), in order to empower service users to take control of their health behaviours and identify their first steps to behaviour change (Barker et al. 2011).

Building self-efficacy and, in turn, self-control, are primary aims of self-management, a process that is advocated in UK health and social care, whereby individuals learn to self-regulate their health conditions. It recognises that providing information about health issues and benefits to change is not enough on its own, and that people should instead be encouraged to manage their own health through, for example, identifying their own solutions, setting goals, and reflecting on their progress. Self-management interventions have been shown to improve clinical indicators, health-related quality of life, self-efficacy, disease knowledge, and control in individuals with chronic conditions, including osteoarthritis, low back pain, and depression (Dineen-Griffin et al. 2019). Moreover, several studies have found self-efficacy a strong predictor of successful self-management (Jang and Yoo 2012; Geng et al. 2018; Do et al. 2015). Other research has highlighted the increased positive impacts of encouraging self-management versus providing standard patient education alone for those with arthritis and asthma (Bodenheimer et al. 2002).

Recognising that building self-efficacy and empowering individuals to take ownership of health behaviours is important for improving outcomes, HCS principles draw from the literature surrounding self-management in chronic conditions, such as asthma and arthritis (Bodenheimer et al. 2002; Barker et al. 2011). However, there is currently little evidence supporting the use of HCS for patients with these chronic conditions. Only one published study has evaluated HCS within the Wessex MECC framework, for clinicians working in roles including sexual health, cardiac health, chronic obstructive pulmonary disease (COPD), weight, smoking, diabetes, and NHS health checks (Lawrence et al. 2020). There is no research to date that has evaluated MECC HCS for others who could benefit greatly from this intervention, such as those with musculoskeletal (MSK) conditions, despite evidence supporting empowering and person-centred self-management interventions for this patient group (Du et al. 2011; Dineen-Griffin et al. 2019; Bodenheimer et al. 2002).

### MECC HCS and musculoskeletal health

MSK conditions, including those affecting the joints (e.g., osteoarthritis), bones (e.g., osteoporosis), spine (e.g., back and neck pain), muscles (e.g., sarcopenia) and multiple body areas, are a growing public health concern and the leading contributor to disability, both globally and in the UK (WHO 2021). Pain is a common symptom of these conditions, reducing quality of life and employment whilst increasing the likelihood of depression, obesity, and other comorbidities (McPhail et al. 2014). There are, however, known risk factors that are associated with the onset and exacerbation of MSK conditions and related pain (Versus Arthritis 2019). Some are amenable to change and could be targeted by public health interventions such as MECC HCS. Anxiety, stress, smoking, and obesity, for example, are significantly associated with increased chronic MSK pain (Louw et al. 2011; Cimmino et al. 2011). Moreover, those with MSK conditions are more likely to be physically inactive than healthy controls (Moseng et al. 2014), often due to the belief that exercise will worsen symptoms, when in fact it can reduce pain and improve function (Smith et al. 2019; Hagen et al. 2012; Barker et al. 2014). Fear-avoidance behaviours such as this have been evidenced to perpetuate pain behaviours and experiences (Sawchuk and Mayer 2008), and provide one explanation for the transition from acute to chronic pain (Zale and Ditre 2015). Other research highlights that pain self-efficacy, or the beliefs someone holds about their ability to participate in daily activities whilst in pain, is associated with pain catastrophising and avoidance (Nicholas 2007), levels of activity, working endurance (Turner et al. 2005), depressive symptoms, and pain severity (Skidmore et al. 2015). Focus on encouraging protective factors (i.e., self-efficacy, exercise, and good dietary quality) is therefore just as important as reducing risk factors (i.e., smoking, stress, obesity) for prevention of MSK disability.

PHE (2019) highlight, in their 5-year MSK Strategic Framework for Prevention, the expansion of physiotherapy services in the UK to support the rising number of service users presenting with MSK conditions. Moreover, they emphasise the importance of implementing evidence-based interventions, including MECC, to promote prevention. Since physiotherapy is the largest of the allied health professions and treats the majority of those with MSK conditions (Chartered Society of Physiotherapy 2013), physiotherapists are uniquely placed to make every contact count with these individuals and could be at the core of promoting MSK health.

There are few localised evaluations of MECC within MSK services in the UK, but available data has been promising in demonstrating an increased number of conversations with patients around physical activity, smoking, weight loss and alcohol (Brace et al. 2022). Moreover, physiotherapist confidence in supporting behaviour change with MSK service users, and referrals to lifestyle services following MECC implementation has been found to increase (Moss and Bancroft 2019). The latter findings have been reported in the Bury and Rochdale Care Organisation, Greater Manchester, where their approach to MECC is being integrated into their physiotherapy service. Barriers to implementation (e.g., lack of time, insufficient training, and lack of confidence) have, however, been reported by practitioners (Cooper-Ryan and Ure 2018). Staff readiness for embedding MECC into everyday practice, due to these issues, was deemed inconsistent across the service. 

Research is warranted to explore the use and perceptions of the Wessex model of MECC HCS, unique in its approach to training and delivery, in order to assess its acceptability within physiotherapy services supporting people with MSK conditions, and identify barriers to implementation. Approaches used in implementation science, such as the Theoretical Domains Framework and COM-B model of behaviour (Cane et al. 2012; Michie et al. 2011) have not yet been used to evaluate MECC HCS within the UK but may be useful in explaining the theoretical underpinnings of implementation (Nilsen 2015). Similarly, little has been done to explore implementation outcomes (i.e., uptake, appropriateness, acceptability, feasibility, sustainability) in relation to MECC HCS. These outcomes can be applied to indicate intervention implementation success and serve as preconditions for desired changes in practice (Proctor et al. 2011).

A recent study conducted in New South Wales, Australia, evaluated the impact of Healthy Conversation Skills training on healthcare professionals’ barriers to having behaviour change conversations, and applied the Theoretical Domains Framework pre- and post-training (Hollis et al. 2021). Quantitative scores for the domains ‘skills’, ‘beliefs about consequences’ and ‘goals’ were significantly higher at 6–10-week follow-up than pre-training, suggesting that Healthy Conversation Skills training addressed some of the barriers perceived by participants for discussing behaviour change. Although these findings are encouraging in highlighting the positive impact of training on perceptions of implementation, no research to date has applied the Theoretical Domains Framework to qualitatively explore barriers and facilitators to MECC HCS, as perceived by those who are already trained in the intervention and delivering it within their organisations.

Utilising the above implementation science approaches in the present study could increase understanding of how successful MECC HCS implementation is currently within the UK, whether it is an acceptable, brief intervention for promoting MSK health, and what could be done to enhance future adoption by physiotherapists supporting people with MSK conditions.

### Study aims

This is the first known study to explore the use and perceptions of the Wessex model of MECC HCS within the UK, i) using validated measures of implementation outcomes, ii) applying the Theoretical Domains Framework to qualitatively investigate contextual factors affecting implementation, and iii) focusing specifically on physiotherapists working with service users with MSK conditions. The following questions are addressed:

1. What is the uptake of MECC HCS by physiotherapists supporting people with MSK conditions, and how does this compare to that of others trained in the brief intervention?
2. How acceptable, appropriate, feasible, and sustainable is MECC HCS for physiotherapists supporting service users with MSK conditions, and how does this compare to perceptions of others trained in the brief intervention?
3. What are the barriers and facilitators to physiotherapists successfully implementing MECC HCS in practice?

## Methods

### Ethics

This study recruited NHS staff only, and therefore did not require approval from the local research ethics committee. The study did, however, receive necessary approvals from the Health Research Authority (HRA), reference 20/HRA/2919, and University of Bath’s Research Ethics Approval Committee for Health (REACH), reference EP 19/20057. Informed consent was obtained from participants at the beginning of the questionnaire.

### Design

This paper forms part of a mixed methods, sequential explanatory study. Consistent with the participant selection model of a sequential explanatory design (Creswell et al. 2003), data collected in this phase of the study were used to; a) develop a general understanding of the current use and perceptions of MECC HCS across individuals within different roles in the UK, and b) generate the purposive sample for the second, qualitative interview phase of the study. The present paper describes findings from an online questionnaire, administered to a range of individuals trained in MECC HCS, which explored the broader use and perceptions of the brief intervention in practice. A subsequent paper (currently in preparation) will report the in-depth, qualitative phase of this study, which focuses specifically on physiotherapists supporting people with MSK conditions, and their experiences of delivering MECC HCS.

### Participants

A range of staff from multiple NHS Trusts, government departments, private sectors, and charities who were trained in the MECC HCS behaviour change intervention were recruited through opportunistic sampling. This was considered the best sampling strategy for the present study due to the challenges associated with random sampling in applied, ‘real-world’ research (Salkind 2010), particularly during the COVID-19 pandemic (see discussion). Recruited participants included both frontline and non-public facing individuals. Those working with service users were in a variety of roles. In addition to physiotherapy, other participants worked in medicine, midwifery, nursing, dentistry, sport/exercise, wellbeing, health improvement, and community work. Non-public facing staff were in roles including senior leadership/management, health development, training, administration, marketing, education, and research. Most participants worked in the Wessex and South West regions of England, where the rollout of MECC HCS has been largest.

### Procedure

Following institutional and external HRA ethical approval, individuals who had previously received training in the MECC HCS Train-the-Trainer programme (2 half-days and 1 full-day training sessions), were identified from a database owned by Health Education England Wessex (the MECC HCS training providers). These individuals were invited, by email, to take part in an online questionnaire exploring their use and perceptions of MECC HCS as a brief intervention. A second email was sent, roughly 5 months later, to remind individuals that the study was still open, giving them further opportunity to participate. Emails were distributed by Health Education England Wessex in order to comply with GDPR; however, contact details of the lead researcher were provided. Other individuals trained in MECC Lite (3-h training session) and the full MECC programme (2 half-day training sessions) were invited to participate via local NHS Research and Development teams, email (via Health Education England Wessex), Twitter, and promotion by organisations (Musculoskeletal Association of Chartered Physiotherapists, Advanced Practice Physiotherapy Network, and Health Education England).

The online questionnaire (accessible on JISC Online Surveys) was developed by the lead researcher and was piloted with ten healthcare professionals before the study period commenced. It consisted of 34 items relating to demographics, use, and perceptions of MECC HCS, taking approximately 15 to 20 minutes to complete. Study recruitment took place over 7 months (August 2020–April 2021). Participants were required to give informed consent online, after reading written information on the nature and purpose of the study. Data were entered into an IMB SPSS database for analysis and saved on a University of Bath password-protected secure server.

### Measures

The online questionnaire consisted of items relating to demographic information, perceived acceptability, appropriateness, feasibility, sustainability, and uptake of MECC HCS. These implementation outcomes are adapted from the growing area of implementation research and indicate implementation processes and success, whilst serving as preconditions for achieving desired changes (Proctor et al. 2011; Damschroder 2020). In order to explore these outcomes in relation to the implementation of MECC HCS, adapted versions of the Acceptability of Intervention Measure (AIM), Intervention Appropriateness Measure (IAM) and Feasibility of Intervention Measure (FIM) were utilised (Weiner et al. 2017). These measures have been supported in the study of intervention implementation, effectiveness, and efficacy as being the first of their kind to be psychometrically assessed, and demonstrate good structural validity, test–retest reliability, and excellent internal consistency (α = 0.85–0.91). They are easy to use, brief, can be applied to many contexts, and are therefore considered pragmatic in nature and useful for a variety of settings (Weiner et al. 2017). The measures have four items per construct and require responses on a 5-point Likert scale ranging from completely disagree to completely agree.

Perceived sustainability was measured using a shortened and adapted version of the Program Sustainability Assessment Tool (PSAT). This consisted of five items focusing on organisational capacity and required responses on a 7-point Likert scale from ‘little to no extent’ to ‘a very great extent’ (Luke et al. 2014). Given the small size of this sub-scale, its developers report that its reliability (internal consistency) is excellent (Cronbach’s alpha = 0.87) and its construct validity moderate (.58). Organisational capacity (having the internal support and resources needed to effectively manage a programme/intervention and its activities) was deemed the most useful construct of sustainability to measure for the purposes of this study, as it is considered a key factor that can influence the successful implementation of MECC (Public Health England, NHS England, and Health Education England 2016). All measures were adapted to include the word ‘MECC HCS’ in order to increase the clarity of each item for participants (e.g., ‘MECC HCS meets my approval’).

Other questions assessed the uptake of MECC HCS. These related to; a) the frequency of MECC HCS used (measured with a 5-point Likert-item, with responses ranging from ‘rarely or never’ to ‘frequently during the day’), b) the proportion of service users seen in a typical week believed to benefit from MECC HCS, and c) the proportion of these service users to which the participant actually delivers MECC HCS. The latter two questions were adapted from a similar previous study (Keyworth et al. 2018a, b).

The final question of the online survey was open-ended and explored barriers and facilitators to delivering MECC HCS.

### Analyses

Mean AIM, IAM, FIM, and PSAT scores were calculated after responses were coded (i.e., 1 = ‘completely disagree’, 2 = ‘disagree’ ,etc.) Higher scores indicate greater perceived acceptability, appropriateness, feasibility, and sustainability. One-way ANOVAs were conducted to explore differences in perceptions and use of MECC HCS between groups. Professional groups were aggregated based on similarity (see results), due to there being very small numbers in some.

Descriptive statistics were used to quantify uptake/use of MECC HCS. Non-parametric, Kruskal–Wallis tests were conducted to compare distributions of frequencies of MECC HCS used between groups.

Reflexive thematic analysis was employed to analyse the final, open-ended questions of the survey, in order to identify common themes regarding the barriers to and facilitators of intervention implementation. Unlike other forms of thematic analysis that are more rigid and structured (such as codebook and coding reliability), reflexive thematic analysis is a fluid and recursive process, whereby the researcher takes an active role in the development of themes (Braun and Clarke 2019). Reflexivity refers to a person’s critical self-awareness of their involvement in the analysis. Reflecting and identifying what is being subjectively assumed from the data are therefore considered good practice for this analysis (Braun and Clarke 2019). The first stage of the analytic process involved data familiarisation. Next, responses were coded and data within each code were collated. Thirdly, initial themes were generated using these codes, reviewed and refined. Themes were defined and given clear names and, next, write-up began. The analysis was conducted deductively, in order to explore how qualitative responses regarding barriers and facilitators to MECC HCS implementation could be linked to components of behaviour (i.e., capability, opportunity, and motivation) and mapped to factors that might influence these behaviours (i.e., environmental context and resources, social influences), thus encouraging or hindering the uptake and delivery of the intervention (Michie et al. 2011; Cane et al. 2012). Furthermore, the researchers were interested in how these factors could be targeted, in order to enhance future rollout and adoption of the intervention. Theories and frameworks applied in behaviour-change implementation science therefore guided the qualitative data analysis process. Themes were mapped to the Theoretical Domains Framework, a framework that is closely linked with Michie et al’s COM-B model of behaviour (2011) and Behaviour Change Wheel (2014) and can be applied in implementation research in order to identify factors that may influence behaviour and inform enhanced future implementation. Patterns of shared experiences of MECC HCS were evident across professional groups; however, for the purpose of the present study and its research questions, the responses of physiotherapists supporting those with MSK conditions are highlighted in the results.

## Results

### Participant characteristics

The total sample (71) included MSK physiotherapists (15), ‘other’ physiotherapists (one brain injury physiotherapist, one myeloma physiotherapist), doctors (one GP, one foundation doctor, one specialist doctor), midwifes/nurses (five), dental professionals (two), sports/exercise professionals (three), wellbeing/community workers (11), health improvement practitioners/managers (four), individuals in other public facing roles (five) and non-public facing roles (21). Job roles were aggregated, due to the very small numbers in several of the original groups (e.g., two participants in ‘dental professionals’, two in ‘other physiotherapists’), in order to explore any differences in means between MSK physiotherapists and those in ‘other primary and secondary healthcare roles’, ‘community roles’ and ‘non-public facing roles’. Primary and secondary healthcare roles included individuals that described themselves as working with ‘patients’ and ‘service users’ within GP clinics, hospitals (outpatients and/or inpatients), and dental practices. Community roles included individuals working with the ‘public’ within schools, colleges, universities, housing services, health and wellbeing services, community groups, and libraries. Non-public facing roles included individuals working with workforces, staff teams, and volunteers. The largest group of participants had completed the MECC HCS ‘Train-the-Trainer’ programme (38%). The fewest number of participants reported using their MECC HCS ‘rarely or never’ (4.2%), whilst the largest number reported using them ‘at least daily’ (26.8%) or ‘a few times per week’ (25.4%). Participant characteristics can be seen below, in Table 1.

**Table 1:** Participant characteristics (*n* = 71)

Variable	*N*	%
Gender		
Female	55	77.5
Male	16	22.5
TOTAL	71	100
Age		
20–30	8	11.3
31–40	21	29.6
41–50	19	26.8
51–60	17	23.9
61–70	6	8.5
TOTAL	71	100
Job role		
Physiotherapist (MSK, chronic pain)	15	21.1
Physiotherapist Other (brain injury, myeloma)	2	2.8
Doctor (GP, F2, specialist)	3	4.2
Midwife/nurse	5	7.0
Dental	2	2.8
Sports/exercise professionals	3	4.2
Wellbeing/community worker	11	15.5
Health improvement practitioner/manager	4	5.6
Other public facing	5	7.0
Non-public facing	21	29.6
TOTAL	71	100
Job role (aggregated groups)		
Physiotherapist (MSK, chronic pain)	15	21.1
Other primary and secondary healthcare roles	16	22.5
Community roles	19	26.8
Non-public facing roles	21	29.6
TOTAL	71	100
Location		
Bath & North East Somerset	6	8.5
Devon	3	4.2
Dorset	8	11.3
Hampshire	33	46.5
IoW	3	4.2
Other (Cambridgeshire, Oxfordshire, Gloucestershire, Somerset, Berkshire, Thames Valley, Surrey, Sussex, Essex, multiple locations, international)	18	25.4
TOTAL	71	100
Level of MECC training		
MECC Lite (1X3 hour session)	18	25.4
MECC Full Programme (2X3 hour sessions)	26	36.6
MECC Train the Trainer	27	38.0
TOTAL	71	100
How frequently do you use your MECC skills?		
Rarely or never	3	4.2
A few times per month	14	19.7
A few times per week	18	25.4
At least daily	19	26.8
Frequently during the day	17	23.9
TOTAL	71	100

### Perceived acceptability, appropriateness, feasibility, and sustainability of MECC HCS

On a scale of 1–5, physiotherapists supporting patients with MSK conditions perceived MECC HCS as being highly acceptable (M = 4.3), highly appropriate (within role M = 4.5, within place of work M = 4.6) and highly feasible (within role M = 4.2, within place of work M = 4.3). On a scale of 1–7, they perceived MECC HCS as being moderately sustainable within their place of work (M = 4.0).

These findings were similar across professional groups (Table 2).

**Table 2:** Perceived acceptability, appropriateness, feasibility, and sustainability of MECC HCS for all job roles

Job Role	Total *n*	Acceptability	Appropriateness (within role)	Appropriateness (within workplace)	Feasibility (within role)	Feasibility (within workplace)	Sustainability
Physio (MSK, chronic pain)	15	M = 4.30 SD = 0.50	M = 4.50 SD = 0.47	M = 4.58 SD = 0.49	M = 4.20 SD = 0.59	M = 4.25 SD = 0.45	M = 4.04 SD = 1.40
Physio other (brain injury, myeloma)	2	M = 4.13 SD = 0.18	M = 4.00 SD = 0.00	M = 4.33 SD = 0.47	M = 4.13 SD = 0.18	M = 4.00 SD = 0.00	M = 4.08 SD = 2.24
Doctor (GP, F2, specialist)	3	M = 4.83 SD = 0.29	M = 4.33 SD = 1.15	M = 4.67 SD = 0.58	M = 4.17 SD = 1.44	M = 4.58 SD = 0.72	M = 2.60 SD = 2.77
Midwife/nurse	5	M = 3.85 SD = 1.08	M = 3.90 SD = 0.80	M = 3.75 SD = 0.94	M = 3.85 SD = 0.29	M = 3.80 SD = 0.21	M = 2.36 SD = 1.08
Dental	2	M = 5.00 SD = 0.00	M = 5.00 SD = 0.00	M = 5.00 SD = 0.00	M = 5.00 SD = 0.00	M = 5.00 SD = 0.00	M = 5.20 SD = 1.13
Sports/exercise professionals	3	M = 4.33 SD = 0.76	M = 4.67 SD = 0.58	M = 4.67 SD = 0.58	M = 4.59 SD = 0.52	M = 4.17 SD-0.76	M = 4.47 SD = 0.61
Wellbeing/community worker	11	M = 4.02 SD = 0.77	M = 4.07 SD = 0.78	M = 4.18 SD = 0.67	M = 3.95 SD = 0.86	M = 3.98 SD = 0.74	M = 4.30 SD = 1.15
Health improvement practitioner/manager	4	M = 4.69 SD = 0.38	M = 4.88 SD = 0.14	M = 4.50 SD = 0.71	M = 4.94 SD = 0.13	M = 4.31 SD = 1.07	M = 5.00 SD = 1.57
Other public facing	5	M = 4.70 SD = 0.45	M = 4.40 SD = 0.55	M = 4.40 SD = 0.55	M = 3.95 SD = 0.62	M = 3.95 SD = 0.62	M = 4.68 SD = 1.61
Non-public facing	21	M = 4.50 SD = 0.53	M = 4.29 SD = 0.73	M = 4.38 SD = 0.77	M = 4.26 SD = 0.52	M = 4.22 SD = 0.66	M = 4.52 SD = 1.85
Total MEAN		M = 4.37 SD = 0.63	M = 4.34 SD = 0.68	M = 4.40 SD = 0.67	M = 4.21 SD = 0.65	M = 4.17 SD = 0.63	M = 4.19 SD = 1.63

In general, MECC HCS was considered to be:

i) highly agreeable and satisfactory (Proctor et al. 2011), reflected by M = 4.4 for all participants on the Acceptability of Intervention Measure
ii) highly relevant and suitable in practice (Proctor et al. 2011), reflected by M = 4.3 (within specific role) and M = 4.4 (within workplace) for all participants on the Intervention Appropriateness Measure
iii) highly practicable (Proctor et al. 2011) within both their role and workplace, reflected by M = 4.2 and M = 4.2 (respectively) for all participants on the Feasibility of Intervention Measure.

The extent to which participants felt they had the internal support and resources to effectively manage MECC HCS and its activities was perceived as moderate, as reflected by M = 4.2 on the ‘Organisational Capacity’ domain of the PSAT. The range of means between professional groups was, notably, largest for this particular measure (2.36 to 5.20), as seen in Table 2.

Professional groups were aggregated (see Table 1). One-way ANOVAs revealed no significant differences in mean scores of perceived MECC HCS acceptability (*p* = .468), appropriateness (*p* = .612 within role, *p* = .454 within workplace), feasibility (*p* = .618 within role, *p* = .721 within workplace) and sustainability (*p* = .083) between physiotherapists supporting people with MSK conditions, and other professionals trained in the intervention.

Further analysis did, however, reveal a significant interaction between; i) perceived sustainability of MECC HCS and the location in which participants worked, and ii) perceived sustainability of MECC HCS and participant age.

Participants working in Devon perceived the sustainability of MECC HCS as being significantly higher (M = 6.5), than those working in the Isle of Wight (M = 2.5), *p* = 0.045, η^2^ = 0.157 (large effect size).

Participants aged 20–30 years old perceived the sustainability of MECC HCS as being significantly higher (M = 5.3) than those aged 41–50 (M = 3.3), *p* < 0.05, η^2^ = 0.009 (large effect size). Those aged 61–70 also perceived MECC HCS sustainability as significantly higher (M = 5.5) than those aged 41–50 (M = 3.3.), *p* = 0.009, η^2^ = 0.182 (large effect size).

### Uptake and use of MECC HCS

Two physiotherapists supporting people with MSK conditions, one within another primary/secondary healthcare role, eight participants within community roles and 13 within non-public facing roles were omitted from the analysis due to incomplete data. Forty-seven responses were included in total. Results are presented in Table 3.

**Table 3:** Use of MECC HCS across professional groups

Job role	‘Approximately how many people do you see/support in a typical week at work?’ (M)	‘Of those you see in a typical week, how many do you think would benefit from MECC HCS?’ (M)	‘Of the service users that you think would benefit from MECC HCS, approximately for what percentage do you deliver MECC HCS?’ (M)
Physiotherapist (MSK, chronic pain)	32	78%	66%
Other primary and secondary healthcare roles	60	62%	51%
Community roles	33	76%	69%
Non-public-facing roles	15	80%	71%

Overall, participants reported that 71% (mean) of people they saw in a typical week would benefit from MECC HCS. Participants reported actually delivering MECC HCS to 63% (mean) of those they believed could benefit.

The proportion of people who, according to participants, would benefit from receiving MECC HCS as a behaviour change intervention ranged from 62% to 80% across aggregated professional groups. The group reporting the lowest percentage of people seen in a typical week with whom MECC HCS would be beneficial were those in primary and secondary healthcare roles. The group reporting the highest percentage believed to benefit were those in non-public facing roles.

The group reporting the highest proportion of people believed to benefit, with whom they actually deliver MECC HCS were participants in non-public facing roles, who reported delivering the intervention to 71% of those they felt would benefit. The lowest proportion (51% of patients) was reported by those in primary and secondary healthcare roles.

### Frequency of using MECC HCS

The median frequency of MECC HCS being used, reported by physiotherapists supporting people with MSK conditions, was 4 (‘at least daily’). Figure 3 shows the median scores for all groups.

Kruskal–Wallis H tests were conducted to explore differences in how often participants reported using their MECC HCS skills (from ‘rarely or never’ to ‘frequently during the day’) between groups. There were no statistically significant differences in frequency of skills used between the six locations (*p* = .300), three levels of training (*p* = .425), two genders (*p* = .723), five age groups (*p* = .567) or four aggregated professional groups (*p* = .292).

### Barriers and facilitators to MECC HCS implementation

Five main themes were developed during reflexive thematic analysis of the final two, open-ended questions of the survey that explored perceived barriers and facilitators to successful MECC HCS implementation. These themes were 1) practical context for facilitating MECC HCS, 2) support from the organisation for facilitating MECC HCS, 3) positive personal perceptions of MECC HCS, 4) patient-related factors, and 5) having the skills to successfully deliver MECC HCS. Each main theme consisted of several subthemes. Quotations taken from physiotherapists specialising in MSK and chronic pain are used to illustrate the themes. However, patterns of shared experiences and perceptions of the brief intervention were evident across groups, suggesting there are universal challenges to MECC HCS implementation that could be targeted on a broader scale. Themes were mapped to six domains on the Theoretical Domains Framework. The most prominent domain was Environmental Context and Resources, followed by Skills (see Table 4). Appendix 2 (Table 5) details all domains of the Theoretical Domains Framework and their descriptions.

**Table 4:** Summary of barriers and facilitators to HCS implementation mapped to six domains on the Theoretical Domains Framework

Barrier	Facilitator	Domain
Theme (i): Practical context for facilitating MECC HCS		
Lack of time	Having time to deliver it	11. Environmental Context and Resources
Limited access to resources	Up to date reminders, reference materials, and reminders about what is available	
The impact of COVID-19		
Theme (ii): Support from organisation for facilitating MECC HCS implementation		
Lack of support and understanding of MECC from leadership	Buy-in from leadership	11. Environmental Context and Resources
Lack of/difficulty training staff	Access to education and training in MECC HCS	
	Support from other colleagues	12. Social influences
Theme (iii): Positive personal perceptions of MECC HCS		
Lack of motivation and ignorance towards MECC HCS	Motivation for MECC HCS and its principles	9. Goals
	Seeing the benefits of MECC HCS	6. Beliefs about Consequences
Theme (iv): Patient-related factors		
Patients being resistant to behaviour change	Patients being open to behaviour change	6. Beliefs about Consequences
A poor relationship with the patient	A good rapport with the patient	3. Social/Professional Role and Identity
Theme (v): Staff ability to successful deliver MECC HCS		
Not being confident or remembering to use the skills	Having and remembering to use the skills	2. Skills
Lack of interpersonal qualities for facilitating MECC HCS delivery	Good interpersonal qualities for facilitating MECC HCS delivery	

#### Practical context for facilitating MECC HCS

Time was highlighted by the majority of participants as both a key barrier and facilitator to delivering MECC HCS. Constraints on time were apparent across professions, but particularly those that were patient facing, and reduced participants’ opportunities to have conversations about behaviour-change-related issues. One physiotherapist mentioned that having time to conduct follow-up conversations was a facilitator to intervention delivery.

*“For me, [a barrier is] time. MSK physio is going through a radical shift… time slots are already condensed from ‘traditional’ physiotherapy contact times.” (Participant 39, MSK physiotherapist).*

*“[A barrier is] restricted time per patient contact.” (Participant 71, MSK physiotherapist).*

*“[A facilitator is] having enough time…to stop everything and talk with the patient.” (Participant 71, MSK physiotherapist).*

*“Time to follow up conversations [is a facilitator].” (Participant 59, MSK physiotherapist).*

Access to resources was another barrier to successful implementation, as many felt that there were a lack of signposting materials and up-to-date information about health-related services. Up-to-date reference materials, posters and reminders about the services and resources available were considered facilitators to delivering MECC HCS.

*“[A facilitator is] knowledge of where to signpost patients. [A barrier is] difficulty in keeping up to date with what is available in the community to signpost patients to i.e., classes and services.” (Participant 19, MSK physiotherapist).*

*“[A facilitator is] up-to-date reference materials. [A barrier is] resources being out of date.” (Participant 22, MSK physiotherapist).*

The impact of COVID-19 was highlighted as a barrier to MECC HCS delivery for participants in both public- and non-public-facing roles. Lockdown measures and remote working were perceived to hinder one’s ability to deliver effective, face to face behaviour change intervention.

*“The challenges of remote working/ COVID restrictions. I myself am currently working remotely, hence I’m not having contact with ‘service users’ or patient…in my opinion, remote working is a barrier to implementing and delivering MECC” (Participant 45, Education Manager).*

*“[A barrier is] COVID and the resulting lockdown.” (Participant 32, Commercial Manager).*

#### Support from the organisation for facilitating MECC HCS implementation

Buy-in from leadership was considered an important facilitator for successful implementation. Management and leadership not understanding the concept of MECC or supporting staff to use it was a barrier.

*“[A facilitator is] support from leadership and consistency within the organisation.” (Participant 58, MSK physiotherapist).*

*“Leadership needs buy-in from the top to enable effective roll-out and implementation.” (Participant 1, Health Development Officer).*

*“[A barrier is] not all managers feel that it is appropriate for their staff to attend [MECC training].” (Participant 49, Clinical Educator).*

Support and promotion from other members of staff was also a perceived facilitator. Colleagues using the same, consistent approach to MECC HCS, and all members of staff being on-board with implementing it were considered advantageous to implementation.

*“[A facilitator is] colleagues also using this method.” (Participant 62, MSK physiotherapist).*

*“[A facilitator is] everyone using [MECC HCS]”. (Participant 40, MSK physiotherapist).*

Finally, access to education and training in MECC HCS, including subsequent opportunities for staff to refresh their skills, was considered a key facilitator to successful implementation. A lack of training in skills to implement MECC, and challenges associated with organising training, were barriers.

*“[A facilitator is] adequate training.” (Participant 62, MSK physiotherapist).*

*“[A facilitator is] offering staff training to remind them.” (Participant 23, MSK physiotherapist).*

*“[A barrier is] lack of/difficulty training up staff.” (Participant 19, MSK physiotherapist).*

#### Positive personal perceptions of MECC HCS

One’s motivation for adopting a MECC HCS approach was highlighted as both a barrier and facilitator to its implementation. Participants across professional groups felt that buy-in from staff themselves and a belief in MECC HCS’s purpose encouraged its implementation.

*“[A facilitator is] clinician’s buying into the principles of MECC, e.g., if one believes and embraces it or not. [A barrier is] ignorance of what it is about.” (Participant 60, MSK physiotherapist).*

*“[A facilitator is] motivation — seeing the value [of MECC HCS] and being committed to training as many people as possible.” (Participant 1, Health Development Officer).*

Seeing the benefits of MECC HCS was also considered a facilitator, since participants were able to see the positive impact healthy conversations could have on those to whom they were delivering the intervention. One physiotherapist who supported people with MSK conditions commented on promoting ownership and self-management of health conditions and behaviours through delivering MECC HCS.

*“Gives patients back the power to self-manage and be responsible for their health.” (Participant 28, MSK physiotherapist).*

*“Seeing the positive impact MECC can have on conversations.” (Participant 9, Library Technical Assistant).*

#### Patient-related factors

Patient receptiveness was both a perceived barrier and facilitator to MECC HCS implementation, across public-facing professional groups. Participants felt that if patients were open and willing to change their behaviours, this encouraged the delivery of the intervention, whereas resistance to change and a lack of willingness to discuss lifestyle behaviours hindered it.

*“[A facilitator is] patients wishing to change their behaviour.” (Participant 62, MSK physiotherapist).*

*“[A barrier is] getting vibes patients are not ready to consider change or discuss lifestyle habits.” (Participant 29, MSK physiotherapist).*

*“[A barrier is] patient preconceived ideas and pre-existing habits.” (Participant 58, MSK physiotherapist).*

The professional relationship between the clinician and patient was also considered to be both a potential barrier or facilitator to intervention implementation for those in public-facing roles.

*“[A facilitator is] having a rapport with patients.” (Participant 29, MSK physiotherapist).*

*“[A facilitator is] building patient rapport.” (Participant 71, MSK physiotherapist).*

*“[A barrier is] a poor physiotherapist-patient relationship.” (Participant 58, MSK physiotherapist).*

#### Having the skills to successfully deliver MECC HCS

Participants across professional groups felt that possessing the relevant skills and remembering to use them was a perceived facilitator to MECC HCS delivery. Participants and/or their colleagues feeling that they neither had the skills nor could remember to use them was highlighted as a barrier.

*“[A facilitator is] having the clinical skills, e.g., communication, listening. [Barriers are] poor communication skills and lacking reflection.” (Participant 60, MSK physiotherapist).*

*“[A facilitator is] the use of social prescribing skills.” (Participant 62, MSK physiotherapist).*

*“[A barrier is] not feeling prepared or comfortable to discuss weight management/smoking/alcohol intake with patients.” (Participant 19, MSK physiotherapist).*

*“People forget.” (Participant 22, MSK physiotherapist).*

*“Staff not confident on how to have those conversations.” (Participant 23, MSK physiotherapist).*

Several participants also considered some personal qualities as facilitating successful implementation of the intervention, whilst a lack of others posed as a hindrance.

*“[A facilitator is] the ability to have an open, honest conversation.” (Participant 58, MSK physiotherapist).*

*“Being empathetic and approachable.” (Participant 52, Health Improvement Practitioner).*

*“[A barrier is] having poor time management.” (Participant 60, MSK physiotherapist).*

## Discussion

The aims of this study were to explore the use of and perceptions of MECC HCS by physiotherapists supporting people with MSK conditions, compared to others who had received the MECC HCS training. Informed by approaches used in implementation science (Nilsen 2015), it is the first known study to quantitatively explore the perceived acceptability, appropriateness, feasibility, sustainability, and uptake of MECC HCS for those already trained in and delivering the intervention within the UK. It is also novel in qualitatively exploring the barriers and facilitators to MECC HCS implementation, as perceived by those trained in it, using the Theoretical Domains Framework and COM-B model of behaviour. Findings are discussed below as they answer each study question.

### What is the uptake of MECC HCS by physiotherapists supporting people with MSK conditions, and how does this compare to that of others trained in the brief intervention?

There were no significant differences in the self-reported frequency of MECC HCS use between professional groups, with physiotherapists supporting patients with MSK conditions reporting that they use their skills at least once a day. Since the majority of these participants reported being trained in MECC HCS more than 1 year ago, these findings are promising in showing that the use of learned skills that facilitate person-centred, empowering health conversations, is prevalent far beyond training. They complement other studies that have evidenced HCS being implemented by health and social-care practitioners in the long-term, 1-year post training (Lawrence et al. 2016; Baird et al. 2014), and suggest that skills might also be sustained by physiotherapists supporting MSK service users in behaviour change.

There were, however, discrepancies between the number of service users that participants felt could benefit from this behaviour-change intervention and the proportion to which they reported actually delivered MECC HCS. Similar findings were evidenced in a study exploring the prevalence of MECC-related practice across the UK, despite many of the healthcare professionals recruited to the study being ideally placed to practice MECC due to their frequent one-to-one contact with service users (Keyworth et al. 2018a, b). Qualitative follow-up research showed that, although seeing its value, clinicians faced a variety of barriers to delivering an opportunistic behaviour-change intervention, including time, workload, perceptions of their professional role, competence, and the environment (Keyworth et al. 2019). Many barriers to successful implementation of opportunistic behaviour-change interventions appear universal for frontline healthcare professionals (Um et al. 2013; Glowacki et al. 2019; Keyworth et al. 2020).

Since physiotherapists in the present study felt that over three-quarters of their patients could benefit from a brief behaviour-change intervention, it is important that uptake of MECC HCS by clinicians is increased. Targeting common barriers in future implementation strategies could enhance further roll-out of MECC HCS in MSK services, help to engage physiotherapists more in its delivery, and increase the number of MSK patients receiving, and potentially benefiting from, the intervention.

### How acceptable, appropriate, feasible, and sustainable is MECC HCS for physiotherapists supporting service users with MSK conditions, and how does this compare to perceptions of others trained in the brief intervention?

MECC HCS was found to be highly acceptable, appropriate, and feasible across health professions. These findings complement studies that have qualitatively explored the acceptability of MECC HCS in trainees (Dewhirst and Speller 2015; Lawrence et al. 2016; Lawrence et al. 2020), but additionally highlight, using reliable and valid quantitative measures, the perceived relevance and practicability of MECC HCS within a range of professions. Since physiotherapists are ideally placed to deliver brief interventions with MSK service users, their high perceived view of the acceptability, appropriateness, and feasibility of HCS MECC is promising for meeting the goals of the NHS and Public Health England (2019) in promoting prevention and self-management in MSK health via opportunistic brief interventions, as MSK conditions continue to be the leading cause of disability in the UK (WHO 2021).

It is notable, however, that scores for perceived sustainability (through organisational capacity) were not as high as for the other three quantitative measures. Our results suggest that participants felt they had only moderate support and resources within their organisation to effectively implement MECC HCS. Moreover, significant differences in mean scores for sustainability suggest that perceptions of organisational capacity for MECC HCS might differ across locations. More research is warranted to explore this further, particularly to establish how the organisational approach to MECC HCS implementation within NHS trusts in different regions might vary, and what can be learnt from those successfully maintaining MECC HCS in practice.

These particular findings indicate that MECC HCS is well received by physiotherapists supporting those with MSK conditions and chronic pain, and it is valued within their profession; however, the extent to which implementation can be maintained due to system-level, organisational factors must be addressed. This is pertinent, as previous research has highlighted both buy-in and support from leadership, and access to resources as being key facilitators to successful MECC implementation (Dewhirst and Speller 2015; Keyworth et al. 2019; Elwell et al. 2014).

### What are the barriers and facilitators to successfully implementing MECC HCS in practice?

Physiotherapists supporting people with MSK conditions highlighted a number of barriers and facilitators to implementing MECC HCS. Possessing the appropriate skills for delivering the intervention was considered important, as was having a good enough rapport with the patient, and patients being open to changing their behaviours. They additionally felt that being motivated to ‘MECC’ and seeing the positive impacts healthy conversations could have on patients enabled successful implementation. One physiotherapist highlighted MECC HCS as helping patients take ownership and self-manage their MSK conditions. Empowering individuals to take control of their health behaviours by building their self-efficacy is a key principle of MECC HCS, and successful self-management has been evidenced as being strongly predicted by self-efficacy (Jang and Yoo 2012; Geng et al. 2018; Do et al. 2015). For those with MSK conditions, perceived lack of control is associated with feelings of helplessness and disadvantageous adaptation to pain (Koleck et al. 2006; Nicassio et al. 1999). Similarly, pain self-efficacy is associated with pain catastrophising and avoidance (Nicholas 2007), depressive symptoms, and severity of pain (Skidmore et al. 2015). Increasing the self-efficacy and thus self-control of individuals with MSK conditions through MECC HCS delivery could have positive impacts on their health and wellbeing, and should be studied further.

Exploring how context affects implementation (Moore et al. 2014), using the Theoretical Domains Framework, showed that participants faced barriers to MECC HCS implementation mostly associated with ‘environmental context and resources’. Our qualitative findings complement our quantitative data in suggesting that organisational and system-level influences should be targeted to improve implementation of MECC HCS. They also support previous studies that highlight environmental and contextual factors as consistent barriers to implementing the intervention (Dewhirst and Speller 2015; Lawrence et al. 2020; Tinati et al. 2012).

‘Environmental Context and Resources’ as a theoretical domain links to one’s ‘physical opportunity’ on the COM-B model of behaviour (Michie et al. 2011; Cane et al. 2012). COM-B theory posits that capability, opportunity, and motivation interact to influence behaviour (Michie et al. 2011). According to this model, healthcare professionals must have the physical and psychological capability (C), the physical and social opportunity (O), and want/need to engage in MECC HCS more than other, competing behaviours (M). Enhancing the capabilities, opportunities, and motivations of physiotherapists to deliver MECC HCS could thus be important for improving its implementation. Keyworth et al. (2019) provide recommendations for utilising behaviour-change techniques (BCTs) to increase delivery of MECC, and complementary research has showed that BCTs are effective in changing the behaviours of healthcare professionals (Keyworth et al. 2018a, b). Restructuring the physical and social environment, and using prompts and cues are BCTs assigned to targeting ‘environmental context and resources’ as a domain (Michie et al. 2011; Cane et al. 2015; Keyworth et al. 2019). In addition to increasing buy-in from leadership and management in order to promote organisational culture change, employing these BCTs, amongst others (for different domains), could be advantageous in future MECC HCS implementation.

## Limitations

The main limitation of the present study was the smaller than planned sample size. COVID-19 had an impact on the running of this research, affecting the capacity of research teams within NHS trusts for supporting with recruitment, and clinicians having the time to engage in the study. All possible avenues to reach those trained in MECC HCS were explored, such as through the ‘Train the Trainer’ network, Twitter, and promotion through organisations such as the Musculoskeletal Association of Chartered Physiotherapists and Advanced Practice Physiotherapy Network, in addition to contacting local public health leads for several regions in the UK. The small sample size means that our quantitative findings cannot be generalised to all public-facing and non-public-facing staff trained in MECC HCS, since there are a substantial number that have now received training but did not complete the online questionnaire. It is also possible that those that did complete the questionnaire were more interested and favourably disposed towards the use of MECC HCS within their practice.

Our sample size did, however, meet the sample size recommendations of Braun and Clarke (2013) for collecting qualitative survey data for thematic analysis as part of a PhD project (> 50 participants). The qualitative component of this study was novel in exploring contextual factors affecting MECC HCS implementation using the Theoretical Domains Framework, and might offer analytical generalisability (Smith 2018) in highlighting physical opportunity as a main barrier to physiotherapists and other healthcare professionals delivering brief behaviour-change interventions.

Future, post-pandemic research should endeavour to reach as many MECC HCS-trained individuals using stratified sampling strategies, in order to evaluate the intervention on a wider scale and to reach sufficient power for exploring quantitative differences between professional groups. Further evaluation of MECC HCS should also include those receiving the brief intervention, in order to assess the extent to which service users find it acceptable and to understand its impact on their health and wellbeing.

## Conclusion

Findings from this study suggest that MECC HCS is an acceptable brief or very brief intervention for physiotherapists supporting people with MSK conditions. The extent to which the intervention is sustainable within organisations must, however, be addressed and buy-in from leadership and management targeted. A focus on this and other barriers to implementation may help close the gap between the number of service users believed to benefit from MECC HCS and those actually receiving the intervention from trained healthcare professionals. Further roll-out of MECC HCS in MSK services may be beneficial for meeting the goals of the NHS and Public Health England in prevention of chronic MSK conditions and promotion of good MSK health.

## Data Availability

Materials and data set are available from the corresponding author upon request.

## Appendix 2 Theoretical Domains Framework

**Table 5:** Theoretical Domains Framework

Domain	Definition (Cane et al. 2012)
Knowledge	An awareness of the existence of something
Skills	An ability or proficiency acquired through practice
Social/Professional Role and Identity	A coherent set of behaviours and displayed personal qualities of an individual in a social or work setting
Beliefs about Capabilities	Acceptance of the truth, reality, or validity about an ability, talent, or facility that a person can put to constructive use
Optimism	The confidence that things will happen for the best or that desired goals will be attained
Beliefs about Consequences	Acceptance of the truth, reality, or validity about outcomes of a behaviour in a given situation
Reinforcement	Increasing the probability of a response by arranging a dependent relationship, or contingency, between the response and a given stimulus
Intentions	A conscious decision to perform a behaviour or a resolve to act in a certain way
Goals	Mental representations of outcomes or end states that an individual wants to achieve
Memory, Attention and Decision Processes	The ability to retain information, focus selectively on aspects of the environment, and choose between two or more alternatives
Environmental Context and Resources	Any circumstance of a person’s situation or environment that discourages or encourages the development of skills and abilities, independence, social competence, and adaptive behaviour
Social Influences	Those interpersonal processes that can cause individuals to change their thoughts, feelings, or behaviours
Emotion	A complex reaction pattern, involving experiential, behavioural, and physiological elements, by which the individual attempts to deal with a personally significant matter or event
Behavioural Regulation	Anything aimed at managing or changing objectively observed or measured actions
